# Long term hypoxia during gestation alters perirenal adipose tissue gene expression in the lamb

**DOI:** 10.1080/21623945.2020.1763726

**Published:** 2020-05-13

**Authors:** Dean A. Myers, Krista Singleton, Kim Hyatt, Kanchan M. Kaushal, Charles A. Ducsay

**Affiliations:** a Department of Obstetrics and Gynecology, University of Oklahoma Health Sciences Center, Oklahoma City, OK, USA; b Lawrence D. Longo M.D. Center for Perinatal Biology, School of Medicine, Loma Linda University, Loma Linda, CA, USA

**Keywords:** UCP-1, sheep, foetus, adipose, hypoxia

## Abstract

We previously reported that following long-term hypoxia (LTH), the ovine foetus exhibits enhanced expression of brown/beige adipose genes. This study was designed to determine if these changes are preserved after birth. Pregnant ewes were divided among three groups, 1) Control, sea level, 2) LTH, high altitude (3,820 m, LTH-HA) from ~ day 40 of gestation through ~14 days post-delivery and 3) LTH from ⁓ day 40 through day 137 of gestation then returned to the laboratory where atory reduced maternal PO_2_ was maintained by nitrogen infusion. Following delivery, lambs remained at sea level (LTH-SL). Perirenal adipose tissue was collected at ~day 14, and qRT-PCR was used to quantify mRNA. Uncoupling protein 1 (UCP-1), PPAR gamma coactivator 1 (PGC1α), and deiodinase-2 (DIO2) mRNA levels were significantly lower in both LTH groups while PR domain containing 16 (PRDM16) levels did not differ. Peroxisome proliferator-activated receptor (PPARγ) was maintained in the LTH-HA group and significantly increased in the LTH-SL group, compared to control. Unlike our previous LTH foetal studies, the brown/beige fat phenotype was rapidly lost by day 14 postpartum compared to control, while PPARγ was maintained. This loss of the brown fat phenotype may promote obesity due to decreased energy expenditure, favouring fat deposition.

## Introduction

Childhood, adolescent, and adult obesity have risen dramatically over the past few decades [1] and obesity prevalence among children and adolescents (age 2–19 years) has nearly tripled since 1980 and is estimated at 17% [2]. Similar to adults, childhood obesity increases risk for hypertension, type II diabetes/insulin resistance, dyslipidemia, and left ventricular hypertrophy. The Bogalusa Heart study reported that overweight children were 4.5 and 2.4 times more likely to have elevated systolic and diastolic blood pressure, respectively [3]. Childhood obesity is nearly irreversible, and one of the most reliable predictors of adult obesity and metabolic disorders [4,5]. While maternal obesity (gestational and pre-gestational obesity) is strongly linked as a causative factor in childhood obesity, it is apparent that not all children born from obese women develop obesity, and a significant population of children from non-obese/diabetic mothers develops childhood obesity [6]. This suggests that other factors during gestation may program the offspring to develop obesity.

In a wide range of species, substantial evidence supports the concept that an adverse intrauterine environment has permanent effects on the foetus leading to increased susceptibility of the offspring to a variety of interrelated metabolic and cardiovascular disorders as adults, including obesity [7–10]. The intrauterine environment’s reprogramming of adipose remains poorly understood, particularly in species such as sheep and humans, where adipose undergoes significant differentiation and expansion during foetal development. Foetal perirenal fat (PRF) is classically considered a ‘brown’ adipose tissue (BAT), expressing the thermogenic mediator, uncoupling protein 1 (UCP1) [11,12] and genes that support UCP1 expression [13,14]. In the late gestation foetus, the PRF BAT phenotype is driven by glucocorticoids and thyroid hormones [15,16]. Unlike actual BAT (e.g., supraclavicular), PRF loses the BAT phenotype post-birth while retaining/expanding the white adipose tissue (WAT) phenotype [17,18]. The loss of the BAT phenotype is not due to apoptosis, but rather the loss of the molecular program of gene expression necessary for BAT function (UCP1, PGC1α, PRDM16, DIO2). Beige or BRITE (‘brown-in-white’) adipocytes have been characterized that reside in WAT of adults.

The abrupt loss of the BAT phenotype of foetal PRF in the early neonatal period [17] suggests that this foetal fat deposit may be more reflective of it being beige fat rather than BAT per se. Alternatively, the PRF may contain populations of both white adipocytes and beige/BRITE adipocytes, and after birth, the white adipocytes expand. In contrast, the beige/BRITE adipocytes lose the phenotypic gene expression of BAT yet continue to reside in the PRF, providing the potential for activation upon proper stimulation. In adult rodents, the molecular program of gene expression supporting BAT function in beige adipocytes is induced by sympathetic nervous system activation as well as via hepatic FGF21 (an endocrine member of the FGF family) [19–22]. This process, termed ‘beiging’, is protective against obesity by lipid mobilization. However, the generation of beige adipocytes during foetal development remains largely unexplored.

One of the factors that may regulate gene expression in foetal adipose tissue is hypoxia. Moderate gestational hypoxia is an unfortunate yet frequent foetal perturbation associated with preeclampsia [23,24], maternal obesity [25], placental insufficiency [26], smoking [27] and, high altitude [28]. Indeed, maternal smoking during pregnancy, which is linked to foetal growth restriction and foetal hypoxia, has also been strongly linked to childhood obesity independent of maternal obesity [29–31]. We reported a significant impact of long-term moderate gestational hypoxia (LTH) on PRF gene expression in the late gestation ovine foetus. We have shown that LTH applied to foetal sheep from early gestation (~40 days gestation; dG) through near-term (~140 dG; term 148dG) results in increased leptin expression in foetal PRF as well as increased plasma leptin, a hallmark of activated adipose tissue [32]. In the near term LTH foetus, we also observed increased expression of UCP1 and genes governing the beige/BAT phenotype: Deiodinase 1 (DIO1), DIO2, 11βhydroxylase-1 (HSD11B1), PPARγ, PGC1α, and PRDM16 in the PRF. The goal of this study was to determine the effects of LTH on BAT/beige adipose gene expression in the early postnatal period during the transition from the BAT/beige adipose phenotypic ‘switch’ to a predominately WAT.

## METHODS

### Animals

All procedures were approved by the Institutional Animal Care and Use Committees (Loma Linda University School of Medicine). Pregnant ewes (n = 8) were maintained at the Barcroft Laboratory White Mountain Research Station (elevation 3,820 m; long-term hypoxia; LTH) from ~40 days of gestation (40 dG). One cohort of ewes (n = 3), were transferred to the Loma Linda University Medical Centre Animal Research Facility (elevation: 346 m) at 137–138 dG (term = 146 dG). Upon arrival, arterial and non-occlusive tracheal catheters were surgically placed as previously described [33–35]. Maternal PO_2_ was maintained at ~60 mmHg by adjusting the flow of humidified nitrogen (N_2_) gas through the maternal tracheal catheter. Approximately 4 days before the expected delivery date, the ewes were moved to a chamber infused with 14–16% oxygen to continue to maintain maternal PO_2_ at ~60 mmHg as previously described [36]. The rationale was to facilitate the ease of delivery in an area larger than the metabolic cart. Following delivery, the ewes and lambs were maintained in room air, and at ~14 days post-birth, lambs were euthanized for tissue collection (LTH-Sea Level: LTH-SL). The second cohort of ewes (n = 5) was maintained at the Barcroft facility and allowed to deliver at the high-altitude facility (LTH-High-altitude: LTH-HA), and the lambs euthanized at ~14 days’ post-birth. Normoxic control ewes (n = 6) were maintained near sea level (~300 m) throughout gestation and allowed to deliver, and the lambs were euthanized at ~14 days of age (Control). All lambs were maintained with the dams after birth until the time of necropsy. Perirenal adipose tissue from lambs was collected from the anterior pole of the kidney and immediately frozen in liquid nitrogen and stored at – 80°C until analysed. There was an approximately even distribution of male and female lambs in each group.

### Quantification of mRNA via real-time PCR

Real-time quantitative reverse-transcription PCR (qRT-PCR) was used to quantify mRNAs for a battery of genes for either the white fat phenotype or brown/beige fat phenotype using previously validated and published methodology [14,34,35]. The primer sequences are illustrated in Table 1. To describe briefly, (detailed methods are available in referenced citations), total RNA was prepared from PRF samples, DNase I treated to remove residual DNA, and cDNA synthesized via reverse transcription using one μg total RNA, with oligo dT as the primer. Quantitative real-time PCR was carried out using 50 ng of cDNA (assumed equal to input RNA) per PCR reaction for all primers except βKlotho (25 ng) and adiponectin (6.125 ng). All PCR was performed for all samples in triplicate. Primer validation included: 1) a single PCR product (identity confirmed by sequencing), 2) dilution curve of cDNA exhibited a slope of 100% ± 10% ‘efficiency’ where 100% = Δ3 Ct/log cDNA input (Ct is the threshold PCR cycle at which fluorescence is detected above baseline), and 3) the melt curve analysis post-PCR must demonstrate one product. Sybr Green (1 X Sybr green master mix; Biorad, Hercules, CA) was utilized as the fluorophore; PCR was performed utilizing a Biorad iCycler equipped with the real-time optical fluorescent detection system. A three-step PCR was used: 95 C for 45 secs, annealing (primer specific ranging between 55–60 C) for 30 secs, and 72°C extension for 30 secs using a total of 35 cycles. Melt curve analysis was conducted on each sample after the final cycle to ensure that a single product was obtained. Cyclophilin was used as the ‘housekeeping’ mRNA, using the identical first-strand cDNA used for quantification of specific mRNAs of interest and in the same PCR run as for the gene of interest to circumvent any between-run variation. We previously reported that cyclophilin and GAPDH were equally efficacious for the internal housekeeping mRNA [32,35,37]. Control PCR for each primer pair and RNA source were: 1) elimination of reverse transcriptase during first-strand cDNA synthesis and 2) no RNA/cDNA in reverse-transcription reaction. For quantification purposes, a synthetic single-stranded DNA standard was used to generate a standard curve (100, 10, 1, 0.1, 0.01, and 0.001 pg of standard DNA) for extrapolation of starting cDNA concentrations per reaction and in the same PCR block as the unknowns. Linear regression was used to quantify starting RNA (cDNA) based on Ct values as extrapolated from the standard curve.

**Table 1.** Primer sets

Gene		Primer sequence	NCBI Access.No.
CYCLO	FW	5-CCATCGTGTGTCAAGGACTTCAT-3	BT020966 (Bt)
	RV	5-CTTGCCATCTAGCCAGGGTCTT-3	
UCP-1	FW	5-CAGTGAAACTCTACAGTGGGCTGC-3	XM 616977 (Bt)
	RV	5-TGGTGAAGAACTCCTGGACAGTATC-3	
Leptin	FW	5-AGGGTCACTGGCTTGGACTTCATC-3	U62123 (Oa)
	RV	5-CGTGGATCTGTTGGTAGATTGCC-3	
Adiponectin	FW	5-ATCAAACTCTGGAACCTCCTATCTAC-3	KJ159213 (Oa)
	RV	5-TTGCATTGCAGGCTCAAG-3	
PPARγ	FW	5-AGGAGAACGATTCGGCTGAAGC-3	NM_181024 (Bt)
	RV	5-AAAGGCGGGGTTGTTGTTGGTC-3	
PPARα	FW	5-TTGACACAGAGATGCCGTTTTG-3	AF229356 (Bt)
	RV	5-TGACGCTTTATCCCCACAGACC-3	
PGC1α	FW	5-GAGATGTGACCACCGAGAATGAG-3	NM_177945 (Bt)
	RV	5-GCTGTTGACAAATGCTCTTCGC-3	
RIP140	FW	5-CGAGGACTTGAAACCAGAGC-3	NM_015092390.1 (Oa)
	RV	5-TCTTAGGGACCATGCAAAGG-3	
PRDM16	FW	5- ACCAAACCCAAAGAGGCCAA-3	GAAI01006087.1 (Oa)
	RV	5- CTGACATCTGGGGGTGGAAG-3	
DIO-2	FW	5-GACTCGGTCATTCTGCTCAAGC-3	NM_001010992 (Bt)
	RV	5-TGCCACTGTTGTCACCTCCTTC-3	
HSD11B1	FW	5-GGGAATCGGAAGAGAAATGGC-3	NM_001009395 (Oa)
	RV	5-GTAGTGGATGTGGTTGAGAATGAGC-3	
HSD11B2	FW	5-TGTGACTCTGGTTTTGGCAACG-3	NM_174642 (Bt)
	RV	5-AGACGAGAAGAACAGCAGGCAC-3	
βKlotho	FW	5-CCTGGGGTGTCACTGAATCT-3	XM_004010055.3 (Oa)
	RV	5-TCAGCCTCACTCCTTCCACT-3	
FGFR1	FW	5-ATCGTGGAGAACGAATACGG-3	XM_015104616.1 (Oa)
	RV	5-TTCACCTCGATGTGCTTCAG-3	

Abbreviations: Bt: Bos Taurus; Oa: Ovis aries; FW: Forward primer; RV: Reverse primer.

Statistics. ANOVA with Tukey’s post hoc analysis was performed comparing all groups. All values are presented as mean ± SEM.

### Western blot analysis of select genes

Cytoplasmic and nuclear protein fractions were isolated from perirenal adipose tissue using the Pierce NE-PER Nuclear and Cytoplasmic Extraction kit (Pierce Biotechnology; Rockford, Illinois) supplemented with Halt Protease Inhibitor Cocktail (100X, Pierce Biotechnology; Rockford, Illinois). Protein concentrations were determined using a Bio-Rad protein assay (Bio-Rad Laboratories; Hercules, California). Trisglycine SDS sample buffer (4X) and 1.25 M DTT was added to each protein extract and the samples boiled for 5 minutes prior to loading on a 4% to 12% Bis-Tris progein gel (Invitrogen; Carlsbad, California). Proteins were transferred to a nitrocellulose membrane (Bio-Rad; Hercules, California) for 2 hours followed by blocking, washing, and probing using the iBind system (Invitrogen; Carlsbad, California). For the samples in which the cytoplasmic protein was electrophoresed, the primary antibodies were *DIO2, adiponectin and β actin (DIO2, Novus, goat primary, 0.5 μg/ml; adiponectin, mouse monoclonal, 1 μg/ml; AbCam: β actin, Sigma, mouse monoclonal 1:20,000)*. For the nuclear protein electrophoresis, *the primary antibodies were PPARγ (Cell Signalling, 1:500, rabbit), PGC1α (Novus 1:250; rabbit) and* HDAC1 (clone 2E10, Upstate; Lake Placid, New York, 0.55 μg/ml). The secondary antibodies for both cytoplasmic and nuclear fractions were horseradish peroxidase–conjugated rabbit anti-goat, anti-rabbit or anti-mouse (Perkin Elmer; Boston, Massachusetts). The blots were visualized by chemiluminescence (Western Lighting Chemiluminescent Reagent Plus, Perkin Elmer; Boston, Massachusetts) and exposed to film (Santa Cruz; Santa Cruz, California). Densitometry was performed on images of film using NIH ImageJ64 (https://imagej.nih.gov/ij/), and results are expressed in arbitrary densitometric units (AU) after correcting for loading using the control housekeeping protein (β-Actin or HDAC1).

## Results

### Lamb data

There were no differences between the animals in the various treatment groups with respect to age or weight at the time of necropsy Table 2). Birthweights were not obtained due to the risk of neonatal separation on maternal-infant bonding and subsequent nursing. However, as we have published, foetal weights at near term (138–142 days’ gestation, term is 146 days gestation) are not different between normoxic and LTH groups [38].

**Table 2.** Distribution of number, age and weight of lambs in each treatment group

Treatment	n	Age at necropsy (Days after birth)	Weight at necropsy (Kg)
Control	6 (3 M, 3 F)	13.0 ± 0.9	6.4 ± 0.4
LTH-HA	5 (3 M, 2 F)	14.4 ± 0.9	6.8 ± 0.3
LTH-SL	3 (1 M, 2 F)	13.7 ± 0.3	7.2 ± 1.1

M = male, F = female. Values for age and weight represent mean ± SEM.

### mRNA

**Figure 1.**
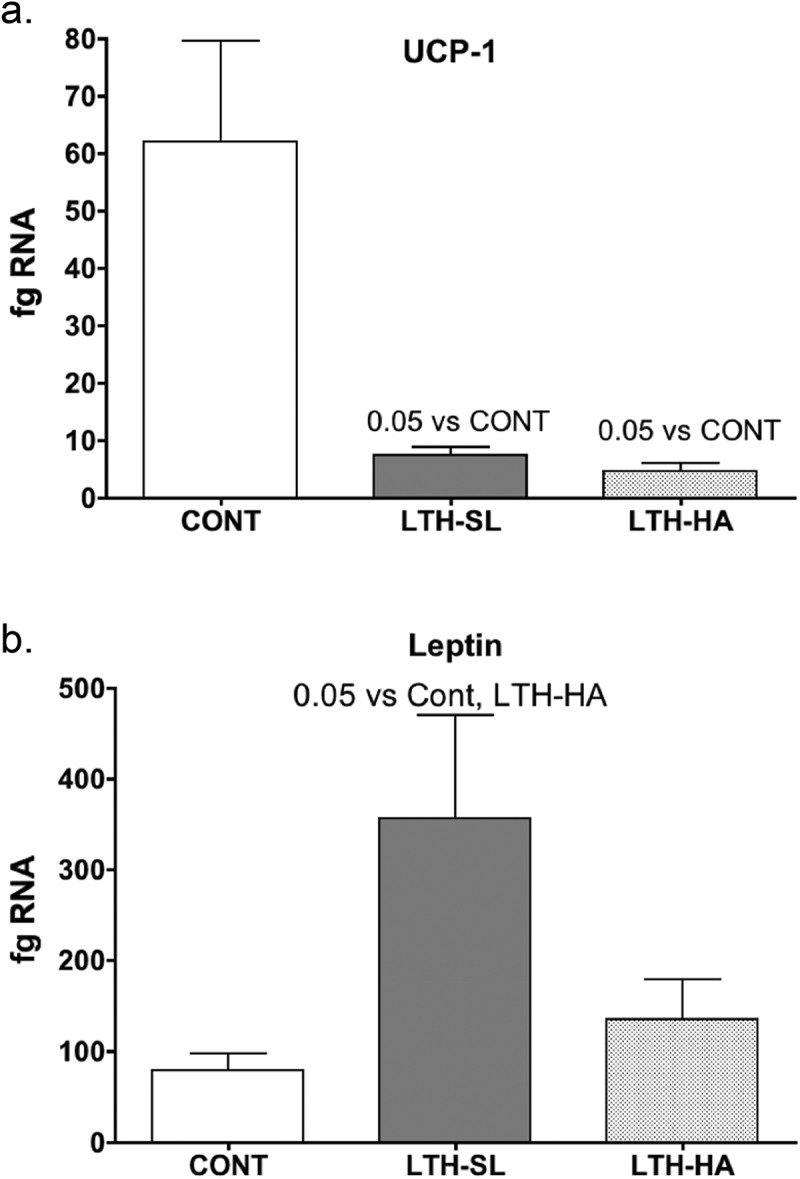
Messenger RNA concentrations (fg mRNA per 50 ng total RNA) for Uncoupling Protein 1 (UCP1) (a) and leptin (b) in perirenal fat from 14-day old control (CONT) lambs gestated and delivered under normoxic conditions, and 14-day old lambs exposed to long term moderate gestational hypoxia in utero (LTH) then maintained post-birth at either high altitude (HA) or near sea level (SL) through 14 days’ post-birth. Lambs exposed to LTH in utero exhibited significantly decreased UCP-1 (a, p < 0.05) in both LTH-HA and LTH-SL lambs. LTH-SL lambs (but not LTH-HA lambs) had elevated mRNA concentration of leptin (b; p < 0.05) compared to normoxic control lambs

**Figure 2.**
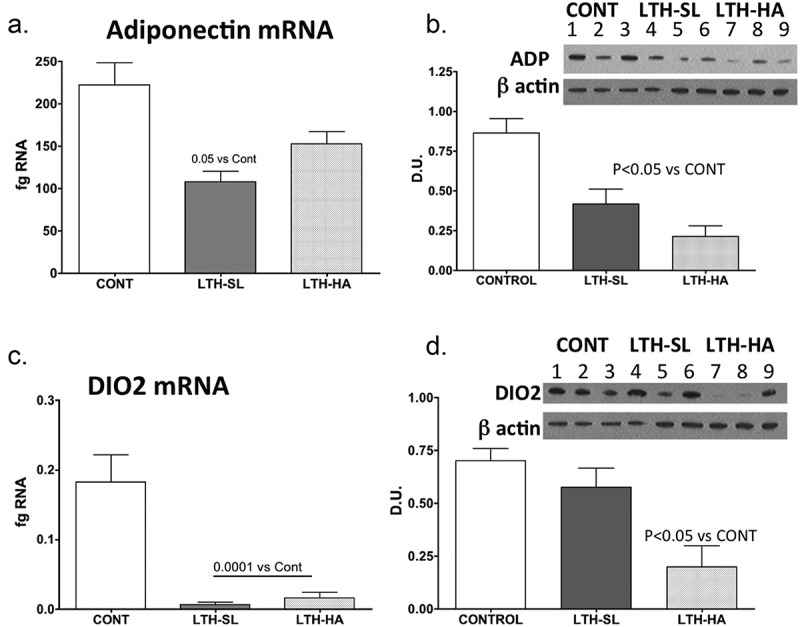
Messenger RNA concentrations (fg mRNA per 50 ng total RNA) and protein levels (Densiometric Units; D.U.) for Adiponectin (a, b) and DIO2 (c, d) in perirenal fat from 14-day old control (CONT) lambs gestated and delivered under normoxic conditions, and 14-day old lambs exposed to long term moderate gestational hypoxia in utero (LTH) then maintained post-birth at either high altitude (HA) or near sea level (SL) through 14 days post-birth. Lambs exposed to LTH in utero had reduced mRNA and protein compared to CONT lambs in the PRF. However, for mRNA, only the LTH-SL lambs did this reach significance. For both LTH cohorts, however, protein was significantly reduced. For DIO2, both LTH-SL and LTH-HA groups exhibited significantly reduced mRNA, while for protein, a reduction was only noted in the LTH-HA group. (lanes 1–3 CONT; Lanes 4–6 LTH-SL; Lanes 7–9 LTH-HA)

**Figure 3.**
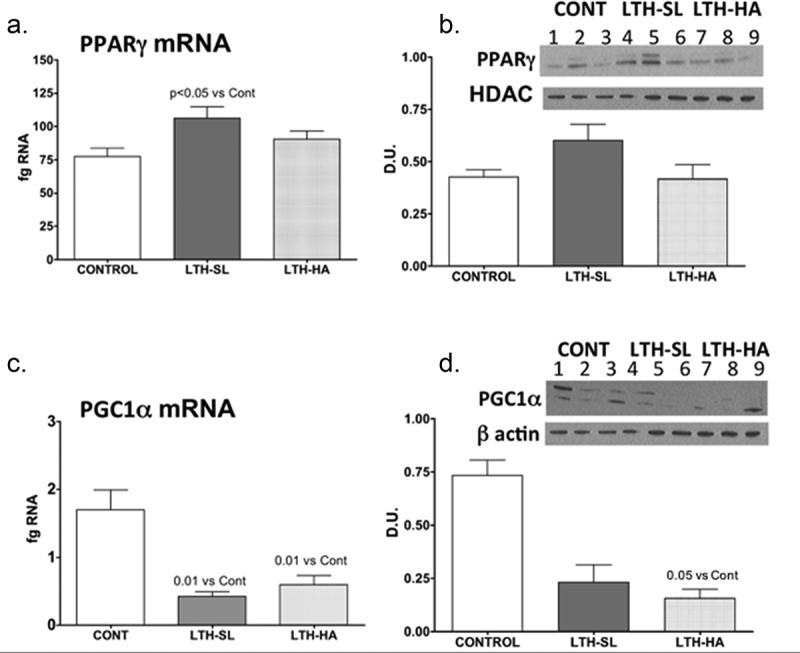
Messenger RNA concentrations (fg mRNA per 50 ng total RNA) and protein levels (Densiometric Units; D.U.) for PPARγ (a, b) and PGC1α (c, d). LTH-SL lambs (but not LTH-HA lambs) had elevated mRNA concentration of PPARγ (a; p < 0.05). A similar pattern was observed for protein (b). Lambs exposed to LTH in utero exhibited significantly decreased mRNA PGC1α (c, p < 0.001) in both LTH-HA and LTH-SL lamb compared to controls. Despite a similar pattern in the LTH groups, only the LTH-HA lambs exhibited a significant decrease in protein compared to controls. (d; p < 0.05; lanes 1–3 CONT; Lanes 4–6 LTH-SL; Lanes 7–9 LTH-HA)

**Figure 4.**
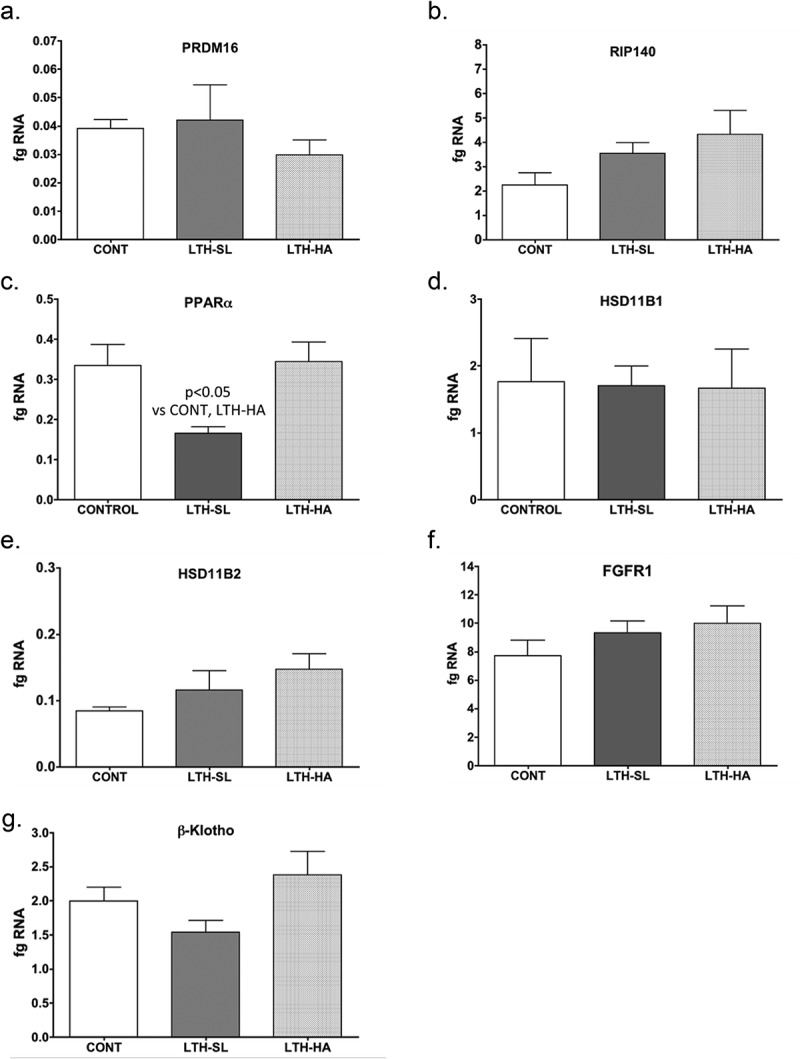
Messenger RNA concentrations (fg mRNA per 50 ng total RNA) for PRDM16 (a), RIP140 (b), PPARα (c), HSD11B1 (d), HSD11B2 (e), FGFR1 (f), and βKlotho (g) in perirenal fat from 14-day old control (CONT) lambs gestated and delivered under normoxic conditions and 14-day old lambs exposed to long term moderate gestational hypoxia in utero (LTH) then maintained post-birth at either high altitude (HA) or near sealevel (SL) through 14 days post-birth. Differences were noted in mRNA for only PPARα, and that was restricted to the LTH-SL cohort (c)

UCP-1 mRNA was significantly lower in both LTH-SL and LTH-HA lamb perirenal adipose compared to control lambs (Figure 1(a)). There were no differences in UCP-1 between LTH-SL and LTH-HA. Leptin mRNA in PRF was significantly higher in LTH-SL lambs but not LTH-HA lamb PRF compared to control lambs (Figure 1(b)). Adiponectin mRNA in perirenal adipose was significantly lower in LTH-SL compared to control (Figure 2(a)). A similar trend was observed in the LTH-HA group but did not reach statistical significance. DIO2 mRNA was significantly lower in both LTH-SL and LTH-HA compared to control (Figure 3(a)); no differences were observed between LTH-SL and LTH-HA for DIO2. PPARγ was significantly elevated in LTH-SL PRF compared to control, with LTH-HA PPARγ mRNA levels not different compared to control and LTH-SL (Figure 3(a)). PGCα was significantly lower in LTH-SL and LTH-HA vs. control (Figure 3(c)), and no differences were found between either LTH group. PRDM16 was not different between any groups (Figure 4(a)). RIP-140 was increased specifically in LTH-HA lambs compared to control lambs (Figure 4(b)); RIP-140 mRNA in LTH-SL lambs was not different between control and either LTH group PRF. PPARα mRNA levels were lower in LTH-SL lambs compared to control and LTH-HA lambs (Figure 4(c)). Neither HSD11B1 nor HSD11B2 showed any difference in mRNA levels in PRF among any of the groups (Figure 4(d,e)). Similarly, mRNA levels for FGFR1 and its receptor binding partner, βKlotho, were not different in PRF of either LTH groups compared to control lambs, and there were no differences between LTH lamb groups (Figure 4(f,g)).

### Protein

We focused on proteins whose mRNA was changed in response to LTH, and of those, we report Western Analysis of results in which the primary antibody could be validated rigorously (absorption control, negative control tissues). Suitable antibodies were not identified for leptin or PPARα. As such, importantly, we were able to analyse PPARγ, PGC1α adiponectin, and DIO2. The former two are primary transcriptional switches for adipogenesis, and DIO2 mediates T3 production, which is critical for brown/beige fat differentiation. Adiponectin plays a vital role in regulating insulin sensitivity. Unfortunately, a suitable antibody for leptin for Western analysis was not found despite trying numerous sources. Although an antibody is available for UCP1 and we have published using it for foetal perirenal fat, the expression of UCP1 in the perinatal lamb fat is ~100 fold lower than foetal, and we could not detect UCP1 via Western analysis despite loading up to 100 ug of protein.

We had perirenal adipose available for 3 lambs per treatment group (3 control, 3 LTH-SL, and 3 LTH-HA). Adiponectin protein was lower in both LTH-HA and LTH-SL PRF compared to control (Figure 2(b)), even though for mRNA, only the LTH-SL group reached significance while the LTH-HA group showed a trend for being lower. For DIO2, protein levels were consistent with mRNA for control and LTH-HA lambs, with LTH-HA DIO2 being less abundant compared to control (Figure 2(d)). For LTH-SL lambs, protein levels were similar to control lambs despite mRNA being lower in the LTH-SL lambs. This indicates that protein turnover may be slower in these lambs at low altitude, lagging behind changes in message. For PPARγ, protein levels largely mirrored that of mRNA, although the low sample number precluded significance being achieved (Figure 3(b)). A similar pattern was observed for PGC1α, where protein and mRNA were in agreement (Figure 3(c,d)).

## Discussion

Despite functioning as a highly thermogenic brown adipose tissue at birth and during the first week, studies in a variety of large animal species show that PRF switches its phenotypic characteristics to that of white adipose tissue over the ensuing first month post-birth. [39–42] Perirenal fat of the ovine foetus undergoes a remarkable phenotypic change during the first week post-birth. As shown by Clarke et. Al [17]., ovine PRF exhibits two distinct periods during this transition period, the first phase during the week one post-birth associated with high expression and activity of UCP-1 and peak thermogenic action at approximately four days’ post-birth. The second phase (days 7 to 30 post-birth) is accompanied by a nearly 3-fold increase in PRF mass, an ~10-fold increase in PRF lipid accumulation, and a dramatic decline in thermogenic capacity and UCP-1 expression.

Indeed, by 14 days’ post-birth, the expression of UCP-1 has established basal values in the neonatal lamb. This decline in the BAT phenotype in the first 7–14 days’ post-birth is accompanied by decreased expression of genes governing UCP-1 expression and the BAT phenotype, including PPARγ, PGC1α, PRDM16, DIO2. Conversely, the expression of genes of the WAT phenotype (leptin, adiponectin, RIP140) is dramatically increased. We previously reported that LTH increases expression of genes of the BAT phenotype in PRF of near-term foetal sheep including UCP-1, PPARγ, PGC1α, PRDM16, DIO2, as well as leptin and adiponectin [14]. In the present study, we addressed the effect of LTH on the post-natal transition of PRF from a BAT to WAT phenotype. Since foetal PRF exhibits characteristics of both WAT and BAT (e.g., white colour in appearance, consists of both unilocular and multilocular adipocytes, and expresses genes consistent with both BAT and WAT), LTH could have lasting effects on the function of this fat deposit, potentially influencing susceptibility to adiposity [40]. Additionally, we compared LTH lambs born and maintained at high altitude for the first two weeks of life (continued mild hypoxia) to those born at sea level and maintained for the first two weeks of life in a normoxic environment.

Previously, we found a profound increase in the expression of genes associated with beiging of the PRF in foetal sheep as the result of being exposed to LTH during gestation. Surprisingly, in the present study, at ~14 days post-birth both UCP-1 and a critical gene that governs the BAT/beige phenotype (PGC1α) were significantly decreased in the LTH lambs regardless of whether they were born at high altitude or at near sea level. While mRNA for UCP-1 was still detectable in control and LTH PRF, protein levels were below the ability to detect using Western analysis despite loading maximal amounts of cytoplasmic protein. This is consistent with loss of expression at the mRNA level compared to that observed in late gestation foetuses and possibly reflects restriction of UCP-1 to select small populations of beige cells remaining in the now predominately white adipose of the PRF in the lambs.

Studies in which PGC1α expression was induced in white adipocytes demonstrated increased expression of UCP-1. At the same time, the loss of PGC1α blunts thermogenic induction via UCP-1 [43,44]. PGC1α is a transcriptional co-activator that mediates its transcriptional actions via interaction with PPARγ. Interestingly, PPARγ was significantly upregulated in PRF of LTH lambs born at sea level but not at high altitude. This observation suggests that continued exposure to a mild hypoxic condition post-birth and/or the thermogenic environment that exists at ~3800 metres modulates PPARγ expression in the PRF. PPARα also promotes adipogenesis, mainly relying on binding to the appropriate co-activator and repressor to govern phenotypic switching between beige and WAT. However, unlike PPARγ PPARα mRNA in PRF was decreased, and only in the LTH-SL group, possibly indicating that the continued hypoxia in the HA group affected keeping this gene repressed. Based on the expression of the other key downstream genes (e.g., leptin, UCP-1), PPARα likely plays a minor role in governing the phenotype of PRF post-birth. PPARγ can also promote white adipogenesis by partnering with RIP140, which represses beige/BAT phenotypic gene expression including UCP-1 [45,46]. RIP140 binds PGC1α directly and antagonizes thermogenic target genes while favouring WAT formation. Deletion of RIP140 leads to the presence of beige adipocytes in WAT [47]. In our study, RIP140 was increased in the LTH lambs born at high altitude but not in those born at sea level. This suggests that continued exposure to the high-altitude environment promotes a WAT phenotype, coupled with the loss in PGC1α in these lambs.

PRDM16 is another gene that governs expression of beige/BAT thermogenic genes, including UCP-1, and also functions as a transcriptional co-activator via several transcription factors including C/EBPβ, PPARγ, PGC1α [48–51]. While a role has been made for PRDM16 in beige/BAT adipocyte thermogenic programming, a decrease in PGC1α may limit its ability to function as such in the LTH lamb since its expression was not different compared to the controls lambs. As such, LTH appears to lead to a post-birth transition in the PRF to a transcriptional regulatory pattern favouring WAT.

The induction of the thermogenic profile in foetal PRF during late gestation in foetal sheep is under hormonal control via both glucocorticoids and thyroid hormone. For instance, the administration of dexamethasone to pregnant ewes during the week before birth increases UCP1 expression in foetal PRF. [52] Similarly, cortisol infusion increases UCP1 expression while foetal adrenalectomy decreases UCP1 expression in ovine foetal PRF [16,53]. Thyroid hormones have also been implicated in regulating BAT, and foetal PRF thermogenic programming including expression of UCP1 [53]. Glucocorticoids induce T3 synthesis and may, therefore exert their effect on the thermogenic maturation of adipose in late-gestation ovine foetal indirectly through the known effects of these steroids on T3 production. In the perinatal transition, DIO2 expression in lamb PRF is approximately half of that at birth by day 7 and by day 30 is nearly 10-fold lower expression compared to birth [13]. Interestingly, although DIO2 mRNA was significantly lower in both LTH groups to an equal level, protein for DIO2 was not significantly lower in the LTH-SL group (although exhibiting a trend). As such, continued hypoxia may provide a stimulus for more rapid turnover in DIO2 in the LTH-HA group and that it takes a longer period post-birth in the LTH-SL lambs for DIO2 expression, mRNA and subsequently protein, to establish its new post-birth baseline. In LTH foetuses, DIO2 expression in PRF was significantly elevated as was HSD11B1, which converts cortisone to cortisol, implicating a role for increases local PRF glucocorticoid and T3 production in the enhanced BAT phenotype of the foetal PRF. In the day 14 lambs, DIO2 expression was significantly lower in the PRF of both LTH lambs born at high altitude and those born at sea level. This indicates that the loss of thyroid hormone signalling in the PRF in response to LTH may play a significant role in the loss of UCP-1 in these lambs post-birth. Whether a decrease in DIO2 (and thus T3) mediates the noted changes in PGC1α and RIP40 or vice versa is unclear at present. HSD11B1 and HSD11B2 expression were similar between LTH lambs and control lambs regardless if born at high altitude or sea level. This is indicative that local control of cortisol synthesis from cortisone or metabolism of cortisol to cortisone in the PRF is not a major player in regulating phenotypic changes of PRF from a BAT/beige adipose to a WAT adipose deposit or the loss of the beige/BAT phenotype observed in the LTH PRF (loss of UCP-1).

We examined two other genes primarily associated with WAT in the lamb PRF. In the late gestation, LTH foetal PRF, both adiponectin and leptin expression was elevated compared to normoxic control lambs. [14] By 14 days’ post-birth, lamb PRF expression of leptin was elevated but only in the LTH lambs born at sea level. Interestingly, in the LTH-HA lambs, leptin expression comparable to control lamb PRF expression. While leptin is a hypoxia-inducible gene [54–56], it appears that continued exposure to high altitude is insufficient to drive increased expression of leptin in PRF in these lambs. It is conceivable that other factors (PPARγ coupled with a loss of PGC1α) may play a more prominent role in leptin induction in the LTH lambs born at low altitude. Post-birth, leptin could play a role in appetite behaviour and the reinforcement of hypothalamic appetitive neurocircuits that could govern appetite. In addition to leptin, a specific effect of LTH coupled with being born and reared in normoxic conditions resulted in significantly decrease adiponectin levels. Again, this may reflect the altered expression of PPARγ in these lambs. Lowered adiponectin is usually associated with increased adiposity/obesity. While we did not measure the amount of PRF in the lambs, LTH foetuses do not exhibit increased adiposity compared to controls near term, and there was not a notable increase in lamb PRF. Adiponectin has been associated with maintaining insulin sensitivity, and lowered adiponectin is associated with insulin resistance/type II diabetes and dyslipidemia as well as cardiovascular pathologies [57]. As such, lowered adiponectin in the LTH-sea level lambs may be a defence mechanism to reduce insulin sensitivity and favour energy storage and may favour adiposity.

Both FGF21 and the sympathetic nervous system (SNS) stimulation have been shown to activate BAT/beige thermogenesis [19–22]. While we did not address the role of the SNS in the changes in the phenotypic profile of lamb PRF in the present study, we did examine receptor systems (FGFR1 and β Klotho, its binding partner) expression in lamb PRF. In foetuses, we found that FGFR1 was the predominant PRF receptor (compared to R3 and R4) and that LTH increased expression of both FGFR1 and β Kotho in the late gestation LTH foetal PRF. However, by day 14 post-birth, we observed no differences in expression of either FGFR1 and βKotho in LTH lambs compared to control. There could still be differences in circulating FGF21 however, between these groups. At present, there are no suitable assays for ovine FGF21.

In closing, we had previously shown that in the ovine foetus, development under conditions of LTH gave rise to the augmented expression of genes critical to the endocrine regulation of adipocyte function and differentiation. More importantly, the expression of UCP1, a gene essential for non-shivering as well as associated regulatory genes were also upregulated by LTH. In marked contrast, in the present study, in the transition from foetal to neonatal life when compared to normoxic controls, LTH lambs demonstrated a significant reduction in UCP1. Interestingly, there were subtle differences in gene expression in the PRF of LTH lambs born and maintained in a mildly hypoxic high altitude mountain environment compared to those born in oxygen replete conditions at near sea level. This implies that continuous hypoxia post-birth may continue to impact the development of the PRF depot. At present, the pathways responsible for this dramatic shift in PRF gene expression observed in response to LTH remained to be determined. However, a wide range of epigenetic mechanisms including DNA methylation, histone modifications, and miRNAs appear likely based on the effect of hypoxia on these pathways [58,59]. Because of the potential role of beige adipose tissue in the defence against obesity it is tempting to speculate that such programmed changes may enhance the propensity towards enhanced adiposity in later life.
